# Antimicrobial Compounds from Eukaryotic Microalgae against Human Pathogens and Diseases in Aquaculture

**DOI:** 10.3390/md14090159

**Published:** 2016-09-02

**Authors:** Charlotte Falaise, Cyrille François, Marie-Agnès Travers, Benjamin Morga, Joël Haure, Réjean Tremblay, François Turcotte, Pamela Pasetto, Romain Gastineau, Yann Hardivillier, Vincent Leignel, Jean-Luc Mouget

**Affiliations:** 1 FR CNRS 3473 IUML Mer-Molécules-Santé (MMS), Université du Maine, Avenue O. Messiaen, Le Mans 72085, France; Charlotte.Falaise@gmail.com (C.F.); gastineauromain@yahoo.fr (R.G.); Yann.Hardivillier@univ-lemans.fr (Y.H.); Vincent.Leignel@univ-lemans.fr (V.L.); 2 Ifremer, SG2M-LGPMM, Laboratoire de Génétique et de Pathologie des Mollusques Marins, Avenue Mus de Loup, La Tremblade 17390, France; Cyrille.Francois@ifremer.fr (C.F.); Marie.Agnes.Travers@ifremer.fr (M.-A.T.); Benjamin.Morga@ifremer.fr (B.M.); Joel.Haure@ifremer.fr (J.H.); 3 Institut des Sciences de la Mer de Rimouski, Université du Québec à Rimouski, 310 des Ursulines, Rimouski, QC G5L 3A1, Canada; Rejean_Tremblay@uqar.ca (R.T.); Francois.Turcotte@uqar.ca (F.T.); 4 UMR CNRS 6283 Institut des Molécules et Matériaux du Mans (IMMM), Université du Maine, Avenue O. Messiaen, Le Mans 72085, France; Pamela.Pasetto@univ-lemans.fr

**Keywords:** biological activity, antimicrobial, antibacterial, antifungal, antiviral, biological activity, *Haslea*, microalgae, sustainable aquaculture

## Abstract

The search for novel compounds of marine origin has increased in the last decades for their application in various areas such as pharmaceutical, human or animal nutrition, cosmetics or bioenergy. In this context of blue technology development, microalgae are of particular interest due to their immense biodiversity and their relatively simple growth needs. In this review, we discuss about the promising use of microalgae and microalgal compounds as sources of natural antibiotics against human pathogens but also about their potential to limit microbial infections in aquaculture. An alternative to conventional antibiotics is needed as the microbial resistance to these drugs is increasing in humans and animals. Furthermore, using natural antibiotics for livestock could meet the consumer demand to avoid chemicals in food, would support a sustainable aquaculture and present the advantage of being environmentally friendly. Using natural and renewable microalgal compounds is still in its early days, but considering the important research development and rapid improvement in culture, extraction and purification processes, the valorization of microalgae will surely extend in the future.

## 1. Introduction

Microalgae are present in almost all ecosystems around the world. They evolved in extreme competitive environments, are largely grazed by highly diverse consumers and exposed to microbial pathogens such as bacteria, viruses and fungi. In order to survive, they had to develop tolerance or defense strategies. The variety of these mechanisms resulted in a high diversity of compounds synthesized from diverse metabolic pathways. It appears that many of these metabolites present very specific chemical structures that are not encountered among terrestrial organisms, and sometimes with a structural complexity that makes often too difficult to reproduce them by hemi-synthesis or complete synthesis [1].

In recent decades, there has been a great trend for research and industrial applications of marine compounds and biotechnology [2,3,4,5,6,7]. Among the large spectrum of marine organisms, microalgae represent a promising resource for blue technologies, due to their rapid growth and usually simple nutriment requirements. Furthermore, many microalgal species are able to grow in saline water or wastewater. This represents an invaluable advantage, considering that freshwater resources are becoming scarce. Microalgae are usually very versatile and able to acclimate to various and changing environments. They offer the opportunity to discover novel molecules or produce known molecules at a lower cost. Due to a tremendous phenotypic plasticity, the nature and amount of their secondary metabolites can be manipulated through control of the culture conditions. Many valuable compounds can be extracted from microalgae, including pigments, lipids, proteins, polysaccharides, vitamins or minerals [8,9,10,11]. If an important research effort in microalgal biotechnology is made to promote the production of biofuels [12,13,14], the variety of compounds generated by microalgae can serve a broad spectrum of applications such as pharmaceuticals, cosmetics, human and animal nutrition, environmental restoration and protection or bioenergy [15,16,17,18,19,20]. Several compounds have shown potent biological activities, such as antioxidants, anticoagulants, anti-inflammatory, antimicrobial or antitumoral [8,9,21,22]. The possible use of these compounds as a source of prebiotics, nutraceuticals, chemopreventive agents or antimicrobial drugs was investigated and has demonstrated promising results [11,23,24,25,26]. Microalgae are also valuable for their production of a diverse range of pigments such as chlorophyll, phycobiliproteins or carotenoids that can be used as dyes for food industry or cosmetics.

The earliest and most common use of microalgae is for aquaculture. They have been used as food source and feed additive to promote the growth of the larvae or the juveniles of various aquatic animals such as finfish, shellfish or crustaceans and can be used for refining process at adult stages [27]. Microalgae can also be supplied to the zooplankton used to feed the larvae of finfish and crustaceans [28]. They are not only essential as a food source but they also permit to improve the quality of aquaculture stock. For instance, the carotenoid astaxanthin, especially abundant in the green microalga *Haematococcus pluvialis*, can be supplied to give or increase color to the flesh of salmon and trout [29]; the blue pigment marennine, produced by the diatom *Haslea ostrearia*, gives a blue-green color to the gills and labial palps of oysters, increasing their market value [30].

Other examples of the microalgae use in aquaculture are the so-called “green-water” and “pseudo green-water” techniques. The “green-water” technique refers to natural phytoplankton populations in outdoor ponds, while the “pseudo green-water” technique relates to the regular addition of selected microalgae. Both techniques allow providing favorable turbidity conditions and/or continuous nutrition to larvae or to the live food [27,31,32]. They have proven multiple benefits over the “clear water” system, for which a series of external filters allows to maintain the water quality, in terms of survival and growth of several animal species [33,34,35,36]. Indeed, “green-water” cultures can help to provide food with high nutritional value with chemical and digestive stimulants, and to improve and stabilize the quality of the culture medium [37,38]. Such culture techniques increase general health and resistance to diseases, thanks not only to a better nutrition [39], but also to the production of antimicrobial compounds by some microalgae. The “green-water” culture, and especially the “pseudo green-water” culture present many advantages as they allow direct supply of nutrients, are easy to manage, environmentally friendly and could lower the use of antibiotics in rearing systems [40].

Considering the remarkable biodiversity of microalgae and the improvement in culture, screening, extraction and purification techniques, it is likely that these microorganisms will represent an important source of new products in the future as part of blue technology. So far, bioactive compounds from cyanobacteria have been more studied than those from eukaryotic microalgae, probably due to their simpler culture methods, and have been the subject of several recent papers [41,42,43,44]. One of the major difficulties of microalgae mass culture is the bacterial contamination [45] while the culture media of cyanobacteria species studies are generally more resistant.

The present work is thus an update of previous works [1,2,23], and a complement to a recent review on freshwater microalgae [46]. It aims to present the available information about the biological activities of eukaryotic microalgae, by focusing on their (i) antibacterial; (ii) antifungal and (iii) antiviral properties, with a special interest on the activity against human pathogens and their potential application in aquaculture against various microbial diseases.

## 2. Antibacterial Activity from Microalgae

### 2.1. Antibacterial Activity from Microalgae against Human Pathogenic Bacteria

The increasing resistance of pathogenic bacteria against a significant number of antibiotics, with consequences for human health, has been a great concern for the past decades and has forced the efforts to find new antibacterial substances [6,47,48]. Some bacteria may infect and cause serious diseases in humans and some others can also provoke foodborne illness inducing moderate to severe nausea, vomiting and diarrhea. Since the pioneer work of Pratt in 1944, which demonstrated the activity of the green alga *Chlorella* against several Gram-positive (G+) and Gram-negative (G−) bacteria [49], the interest for antibacterial compounds from microalgae has been growing. Numerous studies followed to detect compounds with antibacterial activity in microalgae, either to develop new drugs against bacterial infections, or to develop additives for food preservations.

Large screening programs have thus been conducted to assess the potential antibacterial activity of various microalgal extracts against pathogenic and foodborne bacteria. Numerous microalgal species from distinct taxonomical groups originating from various areas [50,51,52], mainly from marine environment [53,54,55,56,57,58], but also from freshwater environment [59,60], or even from the soil [61] were shown to have potent antibacterial activity against both (G+) and (G−) bacteria (Table 1). As screening studies can sometimes include hundreds of different microalgae [51,55,59], Table 1 only presents the microalgae with the highest antibacterial activity or the wider spectrum of activity from these screenings.

It appeared from these studies that the production of antibiotics is largely dependent on the microalgal species [65]. The presence of antibiotic agents can vary widely between different species from the same class, even if some studies presume that the antibacterial activity is predominantly found among the members of the classes Bacillariophyceae and Chrysophyceae [54,55]. The antibacterial activity can also differ within a same species, with ecotypes adapted to different environments [83]. Indeed, the green microalga *Dunaliella* sp. isolated from highly polluted waters proved to be more active against bacteria than its ecotypes isolated from less polluted waters [68].

#### 2.1.1. Toward Improving Extraction Techniques

Beside the microalgal species, the presence of the antibacterial compounds in the microalgal extracts is also highly dependent on the solvent used during the extraction. As the biological activity is rarely found in aqueous extracts [59,79,80], it seems that compounds with an activity against foodborne and human pathogenic bacteria in microalgae are mostly hydrophobic and can be more readily extracted with organic solvents. Some authors found that antibacterial activity was generally found in methanolic extracts [50,59], while some other studies described a better extraction using acetone [79,80], benzene and ethyl acetate [65] or petroleum ether and hexane [66].

Other techniques have been tested to extract bioactive compounds from microalgae, such as supercritical CO_2_, pressurized liquid extraction (PLE) or subcritical water extraction (SWE). These techniques are considered as “greener” than the traditional ones as they do not need large quantities of organic solvents, allow a faster extraction and are more selective toward the compounds of interest [84]. Supercritical CO_2_ method allowed obtaining lipid fractions from *Chaetoceros muelleri* with antibacterial activity against *Staphyloccocus aureus* and *Escherichia coli* [75], while a classic extraction with solvents such as hexane, dichloromethane and methanol did not demonstrate any activity against *E. coli* [74]. PLE and SWE permitted to extract antimicrobial agents from *H. pluvialis* in the red phase with good efficiency [66,69,70]. SWE presents the advantage of not requiring the use of toxic solvents, and low temperatures such as 50 °C can allow a good extraction. SWE could therefore represent an interesting green technique to obtain extracts for natural ingredients, and particularly for the food industry.

#### 2.1.2. Diversity of Antibacterial Compounds Extracted from Microalgae

In some studies, the antibacterial compounds present in the organic extracts were characterized. These bioactive compounds can be pigments, such as phycobiliproteins [72] or chlorophyll derivatives [82,85], but they are most of the time free fatty acids. Short chain fatty acids from *H. pluvialis* [69,70] and long chain fatty acids from *Scenedesmus obliquus* [71] present antibacterial activity against *E. coli* and *S. aureus.* The polyunsaturated fatty acids from *Chlorococcum* strain HS-101 and *Dunaliella primolecta* demonstrated antibacterial activity against the methicillin-resistant *S. aureus* (MRSA), a bacterium causing infections that kill thousands of people per year and which can be highly resistant to conventional antibiotics [64]. Desbois et al. also found fatty acids from the diatom *Phaeodactylum tricornutum* with a very potent antibacterial activity against the MRSA and have characterized three different unsaturated fatty acids involved in the antibacterial activity: the polyunsaturated fatty acid eicosapentaenoic acid (EPA), the monounsaturated fatty acid palmitoleic acid (PA) and the relatively unusual polyunsaturated fatty acid hexadecatrienoic acid (HTA) [77,78]. It has also been observed that the fusiform morphotype of this diatom produced greater levels of EPA, PA and HTA compared to its oval morphotype [86].

In natural environments, fatty acids are released when the microalgal cell loses its integrity and they seem to be involved in an “activated” defense mechanism to protect an algal population against grazing predators [87] and when pathogenic bacteria are around the algae [26]. Moreover, it has previously been shown that fatty acids possess bactericidal properties against a diverse range of bacteria [88]. The exact mechanism of the antibacterial activity of the bioactive compounds is not yet fully elucidated, but it seems that bacterial cellular membranes would be the main site of action [89]. There is some evidence of deleterious effects of fatty acids in the bacterial membrane, causing a cell leakage, a reduction of the nutrient intake and a reduction of the cellular respiration [26]. The antibacterial action of fatty acids may also be mediated by the inhibition of bacterial fatty acid synthesis [87].

These mechanisms could explain why (G+) bacteria are more susceptible to microalgae bioactive compounds than (G−) bacteria. In fact, the bacterial growth inhibition is generally lower when microalgae are tested against (G−) bacteria, and in some cases the tested extracts do not present any bactericidal effect. As examples, the phycobiliproteins and exopolysaccharides from the red microalgae *Porphyridium aerugineum* and *Rhodella reticulata* respectively, were active against the (G+) bacteria *S. aureus* and *Streptoccocus pyogenes* but presented no effect against the (G−) bacteria *E. coli* and *Pseudomonas aeruginosa* [72]. The diatom *P. tricornutum* did not demonstrate antibacterial effect against these two (G−) bacteria either, whereas a good antibacterial activity against the (G+) MRSA was observed [78]. Thus, the difference in sensitivities between bacteria may be due to their complex membrane permeability, making the penetration and the bactericidal action of the compound more difficult.

The potent activity of microalgal compounds, especially free fatty acids, against various bacteria straightens further development in the search for drugs and food preservatives from microalgae. Bacterial resistance to free fatty acids has not been encountered yet [90,91], so their exploitation in medicine deserves to be further investigated [78]. Furthermore, as consumers tend to avoid synthetic additives, microalgae could be good candidates as natural sources against food-borne pathogens.

### 2.2. Use of Microalgae against Pathogenic Bacteria in Aquaculture

Bacteria are nowadays considered as main causes of infections in intensive aquaculture of finfish and shellfish worldwide [92], occurring as well in hatcheries and nurseries as in rear and grow-out ponds. These infections can lead to serious mass mortalities and imply considerable economic losses. A wide range of bacteria are known to infect farmed and wild species with minor to severe consequences on health and survival. Among the important bacterial diseases in finfish we can cite those caused by *Aeromonas* sp. and *Pseudomonas* sp., inducing hemorrhages in many fresh-water species, or *Vibrio* sp. inducing vibrioses in marine fish species [93]. *Vibrio* are able to infect a wide variety of hosts [94] and are also the most common and harmful shrimp pathogenic bacteria [95], with luminous species such as *Vibrio harveyi* involved in mass mortalities in shrimp hatcheries [96]. *Vibrio* sp. have also been demonstrated to be involved in a large number of massive mortalities in bivalve hatcheries [97,98,99,100,101].

Antibiotics have been largely used in intensive aquaculture, as well for finfish [102,103], shrimp [104,105,106,107] or shellfish cultures [108,109]. However, as for humans and terrestrial animals, bacterial resistance in aquaculture is increasing and most antibiotics are less effective [110,111,112]. The presence of drug residues in tissues of aquatic animals and the risk of transferring resistant bacteria to humans have led to a great concern about the use of antibiotics for public health [113]. A few antibiotics are now licensed in aquaculture due to the establishment of strict regulations by the European Conformity (EC) or the Food and Drug Administration (FDA), such as the regulation (EC) No 470/2009 of the European Parliament and the Council of 6 May 2009 laying down Community procedures for the establishment of residue limits of pharmacologically active substances in foodstuffs of animal origin. Various alternative and natural compounds are available today to control aquatic pathogenic bacteria, mainly derived from plants. They present the advantages of decreasing the side effects observed with synthetic antibiotics, being less expensive [114]. In this context, several microalgae species have been investigated for their antibacterial activity in vitro, and in co-culture with pathogenic bacteria, and some studies have also been conducted in vivo with the “green water” technique and using microalgae as food supplements (Table 2).

#### 2.2.1. Benefits of the “Green-Water” Technique against Bacterial Diseases in Aquaculture

The “green water” technique has demonstrated beneficial effects on health, survival rates and resistance of different organisms [32,33,34,35,36,37,39]. Addition of the microalga *Isochrysis galbana* allowed a better viability and a faster grow of the turbot *Scophthalmus maximus* larvae as well as a lower proliferation of opportunistic bacteria [133]. The green microalga *Tetraselmis suecica* first showed good antibacterial activity in vitro against several *Vibrio* species [122] and then proved to reduce in vivo the number of various bacterial species in the water tank of the Atlantic salmon *Salmo salar* [123]. Adding *T. suecica* also reduced the number of *Vibrio* species in broodstock gut, eggs and larvae of the white prawn *Fenneropenaeus indicus*, resulting in improved egg hatching and larval survival [132]. A better survival and physiological conditions of the blue mussel *Mytilus edulis* larvae and the scallop *Placopecten magellanicus* larvae were observed when incubated with supernatant containing marennine, the extracellular blue pigment of the diatom *H. ostrearia*, at concentrations as low as 0.1 mg/L [130]. The use of the diatoms *Skeletonema costatum* and *Chaetoceros calcitrans* to feed *Penaeus monodon* larvae allowed a growth inhibition of the luminous bacteria *Vibrio harveyi* [134]. However, this seemed mainly due to the microbial flora associated with the diatoms rather than their metabolic products. Indeed, the effectiveness of the “green water” culture in preventing bacterial infections and outbreaks can also be attributed to the presence of antibacterial factors in the bacterial, fungal and phytoplankton microbiota associated with this culture technique [124].

Yet, several co-culture experiments with axenic microalgae demonstrated the ability of some species to produce and release compounds with potent activity against pathogenic bacteria. Axenic cultures of *Chlorella minutissima*, *Tetraselmis chui*, *Isochrysis* sp. and *Nannochloropsis* sp. limited the growth of various *Vibrio* species [125], and growth of the luminous bacteria *V. harveyi* highly decreased when co-cultured with pure *Chlorella* sp. [40], *C. calcitrans* or *Nitzschia* sp. [124].

Finally, genetically modified microalgae were also used in vivo. In a recent study, better growth, resistance to bacteria and survival rate were observed in the shrimp postlarvae *P. monodon* fed with “fusant” *Chlorella* and *Dunaliella* [135]. The so-called “fusant” microalgae resulted from the generation of a unique cell through somatic hybridization by fusion of the two protoplasts. This fusion technology is especially used to transfer agronomically useful traits to plants [136] and would allow an improvement of valuable metabolites production from these two microalgae. Another genetically modified microalga tested in “green water” systems is the transgenic line of *Nannochloropsis oculata*, able to produce the bovine antimicrobial lactoferricin (LFB) peptide. These LFB-containing transgenic microalgae were developed and tested as food supplement for the medaka fish *Oryzias latipes* infected with *Vibrio parahaemolyticus*, which displayed a significantly better survival rate after 24 h [137].

#### 2.2.2. Microalgae to Improve the Live-Food Quality

Microalgae also show advantages for the live-food quality [39,138], by reducing the number of associated pathogenic bacteria such as *Vibrio* and allowing a lower risk of transmission to fish larvae. Daily addition of *Dunaliella tertiolecta* to feed the brine shrimp *Artemia franciscana*, considered as an essential part of the live food chain for the culture of fish, conferred a full protection against *Vibrio campbellii* and *Vibrio proteolyticus* [39]. A 4 h incubation of *A. franciscana* with the microalgae *Tetraselmis* sp. resulted in a diminution by 75% of associated bacteria, with a better bacterial diversity and the flora less dominated by *Vibrio alginolyticus* [131]. Similar observations were made by Makridis et al. with incubation of *Artemia* with *T. chui* and *C. minutissima* [129]. These authors proposed that the reduction of *Vibrio* cells in *Artemia* cultures could either be due to compounds released by microalgae or to (G+) associated bacteria.

#### 2.2.3. In Vitro Efficiency of Microalgal Compounds against Marine Bacteria

Several in vitro studies demonstrated the ability of various microalgae to produce antibacterial compounds effective against relevant marine pathogenic bacteria. The whole cells of *Chaetoceros lauderi* [58], supernatant and homogenates of *T. suecica* [122,123] or homogenates from *Stichochrysis immobilis* [121] induced a growth inhibition of various marine bacteria. However, Berland et al. noted that limited attention should be paid to antibacterial activity obtained with broken cells, as in natural conditions substances synthesized have to be released into the water to target another organism [121]. Microalgae producing antibacterial compounds that are not released in the medium cannot indeed be considered gainful in “green water” techniques, but these compounds can be highly useful in the design of novel drugs.

Only a few compounds with activity against marine bacteria have been characterized, such as the polyunsaturated free fatty acid in *P. tricornutum*, identified as eicosapentaenoic acid [78]. An antibacterial activity was also demonstrated in vitro with the blue pigment of the two diatoms *H. ostrearia* and *Haslea karadagensis.* These blue pigments can be observed in the apex of the microalgae (intracellular form) but can also be released in the medium (extracellular form). The purified forms of these pigments [139] have been shown to inhibit the growth of several marine bacteria including *Vibrio* such bacteria belonging to *Vibrio splendidus* clade or *Vibrio aestuarianus* species [30,117,118,119], involved in oysters mass mortality [140,141,142].

**Figure 1:**
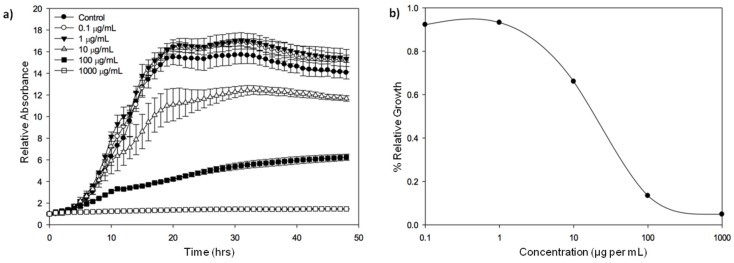
Growth inhibition of *Vibrio tasmaniensis* CIP 107715 by purified marennine, the blue pigment produced by *Haslea ostrearia*. (**a**) *V. tasmaniensis* was grown over night at 25 °C, cells were then washed with sterile water 2 times and adjusted to an OD_600_ = 0.5. Cells were added to wells with marennine in the following concentrations: 0, 0.1, 1, 10, 100 and 1000 μg per mL. Kinetics were run at OD_600_ for 48 h, with measurements taken every 30 min (*n* = 3); (**b**) Relative values were graphed in order to account for the absorbance differences due to the pigment. The effective concentration reducing bacteria growth rate by 50%, EC_50_, was estimated at 19.14 μg per mL (Standard error: 6.73) (original results).

A series of experiments were conducted to assess the spectrum of activity of purified extracellular marennine, the pigment produced by *H. ostrearia*, against pathogenic *Vibrio* species. The growth of *V. splendidus*-related strain (*Vibrio tasmaniensis* LGP32 (CIP107715)) was slowed down when exposed to a concentration of marennine up to 10 μg per mL and seemed totally inhibited with a concentration of 1 mg per mL (Figure 1).

**Figure 2:**
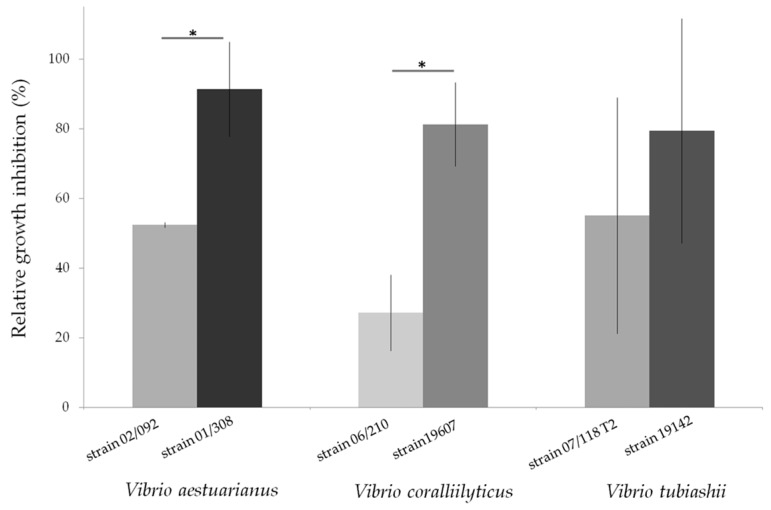
Relative growth inhibition of three *Vibrio* species, *V. aestuarianus*, *V. coralliilyticus*, *V. tubiashii*, after a 48 h exposition to purified extracellular marennine, produced by the diatom *Haslea ostrearia.* Each strain was grown over night in a Mueller-Hinton Broth medium at 22 °C and their concentration was then adjusted to an OD_600_ = 0.1. Bacterial cultures were exposed for 48 h to marennine at a concentration of 100 µg per mL before OD measurement. The relative growth inhibition was assessed in comparison with the growth of the control, not exposed to marennine. Results are means ± SD, for two separate experiments conducted using triplicates. A significant difference of sensitivity (*) between the two *V. aestuarianus* and *V. coralliilyticus* strains was observed (ANOVA statistical test, *p*-value 0.01 and 7 × 10^−4^ respectively). (original results).

In another series of experiments, a comparative approach combining several species and strains was chosen to reflect the diversity of strains within species. The sensitivity of various strains recognized for their virulence, of *V. aestuarianus* [143], *Vibrio coralliilyticus* [144] and *Vibrio tubiashii* [145] to extracellular marennine purified as previously described [139] was assessed by exposing the bacteria to marennine concentration ranging from 1 to 100 μg per mL. For all the three species, after 48 h, the higher the marennine concentration, the higher was the bacterial growth inhibition (data not shown), confirming the antibacterial activity of the pigment produced by the diatom *H. ostrearia*. Moreover, when comparing different ecotypes within a same *Vibrio* species, the sensitivity of the strains exposed to marennine could be significantly different (Figure 2). It can be noted that the strains coming from collections, used to describe the species and often isolated besides mortality events, seemed more sensitive to marennine comparing to virulent strains. Complementary experiments are under way in order to confirm and precise the biological effect of marennine on these *Vibrio* species and to highlight the variability in sensitivity of different strains within a same species.

#### 2.2.4. Interactions between Microalgae and Marine Bacteria

The antibacterial mechanisms of action of microalgae are still unclear and the bioactive compounds released by the cells could either be bactericidal or prevent the bacterial multiplication. The very rapid growth inhibition of various *Vibrio* species in co-culture with *Chlorella* sp., *Isochrysis* sp. or *Nannochloropsis* sp. with no recovery after few days [40,125] allows considering a bactericidal action of the extracellular substances produce by some microalgae [126]. Austin et al. also observed a prompt inhibition of several *Vibrio* species by *T. suecica* in vitro and noticed a very rapid decrease in bacterial mobility with an elongation and vacuolisation of the cells in less than 20 min. Though, a reduction of the inhibitory activity was observed after only 5 h. It was suggested that the bioactive substance could have been denatured or adsorbed by some bacterial cells, allowing others to grow [122,123].

In some cases, the antibacterial activity of microalgae can be induced by the presence of bacteria in the vicinity of the microalgae, or can be constitutive and always present in the algal culture medium [61]. The constitutive production of antibacterial exometabolites by some microalgae was highlighted with the growth diminution of *Listeria monocytogenes* in co-culture with the cell-free culture media of *S. costatum* [127].

Microalgae can influence marine bacteria in different ways. They can either inhibit or stimulate bacterial growth, or have no apparent effect, depending on the target bacteria [121,128]. As an example, the diatom *S. costatum* inhibits the growth of *Pseudomonas* and *Vibrio* in co-culture, but enhances the growth of *Flavobacterium* [128]. The production of antibacterial compounds by microalgae such as lipids or fatty acids varies according to the taxonomic group, the growth conditions, the available nutrients and their concentration in the medium, the light intensity, the temperature or the pH. The development stage of the algal culture is also highly significant as it is assumed that various secondary metabolites are produced and released in the medium at different growth phases [1]. Terekhova et al. showed that only the exometabolites produced by *S. costatum* during the middle steady-state growth phase presented an antibacterial activity against *L. monocytogenes* while compounds released during the exponential growth phase had no effect on these bacteria [127]. Cooper et al. have also demonstrated the direct relation between cell growth phase and antibacterial activity, and showed that *P. tricornutum* presented a better activity against a wide spectrum of marine bacteria during the exponential growth phase compared to the stationary phase, while the reverse relationship was found for *S. costatum* [81].

## 3. Antifungal Activity from Microalgae

### 3.1. Antifungal Activity from Microalgae against Human Pathogens

The search for antifungal compounds from microalgae started much later than screening for antibacterial activity. As a matter of fact, fungi have been considered as harmful human pathogens since the 1970s, when mortality induced by fungal infections and the frequency of nosocomial mycoses increased in hospitalized patients. Increase of fungal infections was mainly due to therapies that depress patients’ immune system such as the use of intensive and aggressive chemotherapy regimens, the expansion of organ transplant programs and the spread of the AIDS epidemic. [146,147]. The incidence of invasive aspergillosis (induced by *Aspergillus* species), and the number of cases of candidemia (an infection and a disease caused by *Candida* species), have been rising inexorably from that time [148,149]. The growing use of antifungal agents in recent years has led to the development of drug resistance [150,151]. Thus, there is a need for novel drugs and several studies were recently conducted to find fungicide activity from natural marine products against human pathogenic fungi [152,153,154,155,156,157], including antifungal agents from microalgae (Table 3).

There are fewer screening activities for microalgal fungicides than for bactericides, and most of the studies focus not only on the antifungal activity but also on antibacterial activities [50,51,58,60,63,65,72,80,117,158,159]. As for antibacterial activity, antifungal activity varies widely depending on microalgal species, type of solvent used to extract the compound and the microorganism tested. It does not seem to be a taxonomic trend for the antifungal activity, and the capability to produce antifungal compounds would have evolved independently of phylogenetic relationship in microalgae [50]. However, Pesando et al. noticed a significant activity of the genus *Chaetoceros* [160] and Kellam et al. also indicated that marine microalgae showed more potential in the search for new antifungal agents than freshwater species [161].

As illustrated in Table 3, several antifungal compounds from various microalgae have been characterized. Polysaccharides with high molecular weight were identified in the diatom *C. lauderi*. They presented a large spectrum of activity against dermatophytes, moulds and phyto-fungi, but no activity was detected against the yeasts tested [58,162,163]. Gambieric acids from the dinoflagellate *Gambierdiscus toxicus* also had an antifungal activity against several dermatophytes and moulds but showed no activity against yeasts like *Candida albicans* or *Saccharomyces cerevisiae* [165]. The diatom *Thalassiothrix frauenfeldii* was meanwhile active against yeasts and moulds but not against dermathophytes [80]. Other compounds such as pigments like beta-carotene, chlorophyll-a and chlorophyll-b from *Chlorococcum humicola* [65], or phycobiliproteins from *Porphyridium aerugineum* have also demonstrated antifungal activities [72]. The polyene-polyhydroxy metabolites amphidinols were extracted from the dinoflagellate *Amphidinium klebsii* [168,169]. Polyenes are metabolites known for having a potent antifungal activity as they target the biosynthetic pathway of ergosterols, found in fungi membranes [150]. These few results illustrate that the search for novel antifungal compounds from microalgae has not been greatly developed so far, although an increasing number of fungi display drug resistance phenomenon.

### 3.2. Potential Use of Microalgae against Fungal Diseases in Aquaculture

An increase in fungal infections has been observed in the last few decades with the modernization and intensification of aquaculture at an industrial scale, resulting in huge losses for aquaculture industries. Fungal infections in aquaculture may cause severe diseases and mortality events leading to economic losses. They are often considered secondary to other factors or pathogens such as consequences of water quality problems, fish trauma by rough handling or temperature shock, bacterial diseases or parasites [170]. Several fungi are known to induce diseases by developing in the skin and the gills of the infected fish or in eggs, or by producing toxins [171]. Indeed, mycotoxins can provoke many disorders and can accumulate in fish tissues, representing a risk for public health [172].

Chemicals used to treat infected animals are limited and, due to the increasing resistance of fungi against conventional drugs, environmental legislations and consumer's safety, the alternative “herbal formulations” and alternative safe and cheap methods have become of renewed interest [173]. Studies have been conducted in order to find new antimycotics of natural origin such as plant extracts [174,175,176] or essential oils [177,178], which should have no harmful effect on fish, fish eggs, human health and ecosystems.

So far, very few studies have been conducted to assess the antifungal activity from microalgae against pathogenic fungi in aquaculture systems (Table 3). Gastineau et al. have demonstrated in vitro the antifungal activity of the pigment produced by the diatom *H. karadagensis* against three marine fungal species *Corollospora maritima*, *Dendryphiella salina*, *Lulworthia* sp., which can be involved in the phenomenon of biofouling [117]. Organic extracts of the green microalgae *Chlorella pyrenoidosa* and *Scenedesmus quadricauda* have demonstrated antifungal activity against *Fusarium monofiliform* [63] reported of causing black gill disease in shrimps [179]. This result is of great interest as *Fusarium* sp. were recently shown to produce toxins that accumulate in fish [180]. *Aspergillus fumigatus* is also a fungus susceptible to produce toxins and it thus represents a potential contaminant for seafood, particularly for marine bivalves [181]. It can be inhibited by compounds produced by *C. lauderi* [58], *G. toxicus* [165], and *Chlorella vulgaris* [62].

More generally, fungi from the taxa *Fusarium* and *Aspergillus* are found in many countries in water, sediments and marine invertebrates, and their presence in shellfish farming areas evidences the necessity to pay attention to shellfish contamination by such fungi [182]. Furthermore, reports of fungi causing deleterious effects are frequently related [183,184], which encourage a larger screening of microalgae for the production of novel antifungal compounds.

## 4. Antiviral Activity from Microalgae

### 4.1. Antiviral Activity from Microalgae against Pathogenic Human Viruses

Viral pathogens are the leading cause of human diseases and mortality worldwide. Treatments to block the entry of the virus or its replication directly are difficult to design, as they can have adverse effects on the infected host cells [185]. Thus, treatments against diseases caused by viruses are limited, and resistance to these available treatments demonstrate the need for new medicines [186]. Many drugs exhibiting selective inhibition of mammalian originate from synthetic organic chemicals or from natural products, for instance secondary metabolites in plants [185]. Along with the development of “blue” technology and extraction improvement, there is a growing interest in marine-derived antiviral compounds. Thus, thousands of compounds from various marine organisms such as algae, bacteria, fungi, marine invertebrates or sponges have been screened [153,186,187,188] and some of them have demonstrated antiviral activities and are commercially available.

Potential antiviral activity from algal compounds has been first demonstrated in the 1950s by Gerber et al. who observed that the polysaccharides extracted from *Gelidium cartilagenium* afforded protection for embryonated eggs against influenza B and mumps viruses [189], and the first brood-based studies of seaweeds for their antiviral substances started in the 1970s [190]. Screenings for antiviral compounds from macroalgae are still predominant [191,192,193,194,195], but the interest for antiviral compounds from microalgae and cyanobacteria rapidly increased as they also present relevant antiviral activities and are easier to culture. Cyanobacteria are promising sources of antiviral compounds, and their simple growth needs make them good candidates for the production of antiviral agents at an industrial scale [44,196]. The sulphated polysaccharide isolated from the cyanobacteria *Spirulina platensis*, named spirulan, has demonstrated potent antiviral activity against the herpes simplex virus type 1 (HSV-1) and also against the human immunodeficiency virus type 1 (HIV-1) [197]. A spirulan-like molecule isolated from *Arthrospira platensis* has also been indicated to possess antiviral activities against these two viruses, with absence of cytotoxic effects [198].

Several studies have been conducted to test microalgae compounds against pathogenic human viruses (Table 4). Antiviral compounds extracted from microalgae are mainly polysaccharides. Their mechanisms of action against viruses are not fully understood but it seems that they can inhibit different stages of the viral infection, such as the adhesion, the penetration or the replication. Polysaccharides have gained interest in the biomedical and pharmaceutical industries as they are easily available in nature and most of them are nontoxic, safe, biodegradable and biocompatible [199]. However, numerous polysaccharides with antiviral activities were not developed for clinical use. The reasons are probably the very high molecular weights of some polysaccharides, preventing them to pass through the different barriers of the body and the incapacity of enzymes to digest these large and complex molecules, leading to their accumulation in the body and to potential cytotoxic effects [200].

Some sulphated polysaccharides present a broad antiviral spectrum against enveloped viruses. Naviculan, extracted from the diatom *Navicula directa*, or A1 and A2 extracted from *Cochlodinium polykrikoides* demonstrated to potent antiviral activity against several enveloped viruses such as HIV-1, HSV-1 or influenza virus type A (IFV-A) [210,211]. The sulphated polysaccharide p-KG03 extracted from *Gyrodinium impudicum* did not demonstrate antiviral activity against HSV-1 and HSV-2, but presented a good activity against the encephalomyocarditis RNA virus (EMCV) [211], and against several strains of influenza viruses with efficiency comparable to some existing drugs [212]. Antiviral activities of microalgae against HSV type 1 and 2 are the most studied [117,118,200,201,202,203,206,208,209,210]. More than one third of the world population is affected by HSV-1 or HSV-2, infections that cause contagious diseases such as oral and genital herpes [187]. The efficiency and the low toxicity of some of the microalgal compounds tested attest their advantageous use as antiviral agents. They could help to control viral diseases occurring in humans, but also in animal species with economic value.

### 4.2. Potential Use of Microalgae against Viruses in Aquaculture

Aquaculture production undergoes numerous viral diseases, which can affect organism health and survival rates, and can sometimes lead to mass mortality. Viral diseases are spreading and are consequently important limiting factors for the expansion of aquaculture. Many different viruses, from various virus families, are known to infect farmed species, such as finfish, crustaceans or molluscs [213,214,215]. As a few relevant examples, we can cite the infectious pancreatic necrosis virus (IPNV), isolated from a very wide host range among finfish [215], the yellow head virus (YHV) and the white spot syndrome virus (WSSV) causing important losses in shrimp culture, or the ostreid herpesvirus-1 (OsHV-1) leading to high mortality in marine bivalves [216] and which can be transmitted between different bivalve species [217]. It appears that diseases induced by RNA viruses are the highest cause of ecological and socio-economic impacts in European farmed finfish [218]. Viruses in aquaculture species are either established for decades or are newly emerging because of the intensification of farming practices that facilitates rapid transmission of diseases.

Viral diseases in aquaculture are challenging to manage. They are difficult to treat directly and a few, if any, efficacious treatments are available other than destroying all organisms in infected farms and avoiding their movements to and from infected areas. In some particular cases, vaccination is used in farmed finfish, mainly to treat trout and salmon [218,219]. The vaccination issue did not arise in invertebrates, as it was widely assumed that, unlike vertebrates, they do not possess the capacity to develop long-term acquired immunity. Nevertheless, there are evidences for specific immune memory in some invertebrates [220] such as crustaceans [221], and several studies have demonstrated the antiviral protection of shrimp by “vaccination” [222,223]. A recent work has also demonstrated the presence of an antiviral system in oysters after injection of a synthetic viral analogue (Poly I:C) against OsHV-1. This immune response showed similarity with the vertebrate interferon response pathway [224]. The lack of marine mollusc cell culture not only restricts virus isolation capacities and subsequent characterization work, but also limits investigations on host-virus interaction. However, recent progress has been made with the development of stem cells in the cupped oysters *Crassostrea gigas* [225].

Plant and herbal extracts with activity against viral diseases in aquaculture production have recently been reviewed by Sivasankar et al. [226]. Some extracts have already been successfully tested in vivo, such as the plant extract of *Cyanodon dactylon* against the WSS virus of the shrimp *P. monodon* [227]. Plant extracts, acting as immunostimulants, have the advantage of being easily delivered by oral administration and may be eco-friendly as they are biodegradable.

In contrast, very few studies have been conducted to assess the antiviral activity of microalgae against viruses in aquaculture. Some polysaccharide extracts of various microalgae have been tested against the viral hemorrhagic septicaemia virus (VHSV) [228], a virus of economic importance afflicting over 50 species of fresh water and marine fish including salmonid fish [215]. Endocellular extracts of *Porphyridium cruentum*, *D. tertiolecta*, *Ellipsoidon* sp., *Isochrysis galbana* var. *Tiso* and *Chlorella autotrophica* inhibited the viral infection of VHSV in vitro in epithelioma papulosum cyprinid (EPC) cells. Concentrations lower than 2 μg of extracts per mL of *P. cruentum* and *D. tertiolecta* were sufficient to detect an antiviral activity. Exocellular extracts of these algal species were also able to inhibit the viral infection, except for *I. galbana* var. *Tiso.* This study has also demonstrated that there is no correlation between the content of sulphated polysaccharides of each microalga and its capacity to inhibit the viral replication. Thus, the observed antiviral effects would be due to different polysaccharide molecular species with differences in molecular size.

A higher resistance to the WSSV was observed in the tiger shrimp *P. menodon* reared in a “green water” system using commercially available extracts of *Dunaliella salina* [35]. Culture bath treatments with the microalga *C. minutissima* significantly reduced the mortality of *Epinephelus marginatus* showing signs of Viral Encephalopathy and Retinopathy (VER) [229]. These results indicate that the control of the disease is probably due to the antiviral effect of *C. minutissima* cultures, which thus needs to be further investigated using in vitro testing of water-soluble algal extracts.

Viral diseases are increasingly spreading, causing great economic losses for aquaculture industries. No effective treatment has yet been developed, as vaccination possibility is limited and chemical drugs are gradually avoided because of their toxicity and potential residue accumulation. Plant and herbal extracts have shown potent antiviral activity against several pathogenic viruses in aquaculture. They could be promising candidates for antiviral agents as part of an environmentally friendly and sustainable aquaculture, although their production and delivery processes could be limiting factors. In this context, the use of microalgae as a source of antiviral agents should be further studied, as water-soluble compounds present in algal supernatants could be a valuable alternative.

## 5. Conclusions and Final Remarks

The diversity of microalgae is immense, with species, genera or even classes being discovered every year. On the estimated millions of existing species, about 30,000 have been described, but only a dozen are cultivated in a large scale for biotechnological applications. The main obstacle for their commercial exploitation remains the production cost, but it should be bypassed by the optimization of mass culturing conditions. Microalgae are promising source of high-value products, and their application as antimicrobials is only in its onset. The development of novel drugs with no microbial resistance and the use of environmentally friendly antibiotics in a context of sustainable aquaculture are needed. The efficiency of various microalgal compounds against human or aquatic pathogens is very encouraging and there is no doubt that their exploitation and application will expand.

## Figures and Tables

**Table 1:** Antibacterial activity from microalgae against human pathogenic bacteria.

Microalgae Species	Antibacterial Compound/Fraction	(G+) Bacteria Growth Inhibition	(G−) Bacteria Growth Inhibition	References
**Green microalgae**				
*Chlamydomonas reinhardtii*	Aqueous or methanolic and exanolic extracts	*Bacillus subtilis*, *Staphylococcus aureus*, *Staphylococcus epidermidis*	*Escherichia coli*, *Pseudomonas aeruginosa*, *Salmonella typhi*	[62]
*Chlorella minutissima*	Ethanolic extracts	*S. aureus*	*E. coli*, *P. aeruginosa*	[51]
*Chlorella pyrenoidosa*	Various organic solvent extracts	*B. subtilis*, *S. aureus*	*E. coli*, *P. aeruginosa*	[63]
*Chlorella vulgaris*	Chlorellin	*B. subtilis*, *S. aureus*, *Streptococcus pyogenes*	*E. coli*, *P. aeruginosa*	[49]
*Chlorella vulgaris*	Aqueous or methanolic and hexanolic extracts	*B. subtilis*, *S. aureus*, *S. epidermidis*	*E. coli*, *P. aeruginosa*, *S. typhi*	[62]
*Chlorococcum* HS-101	alpha-linolenic acid	*B. subtilis*, *Bacillus cereus*, *S. aureus*, MRSA	*Enterobacter aerogenes*	[53,56,64]
*Chlorococcum humicola*	Various organic solvent extracts and purified pigments (carotenoid, chlorophyll)	*B. subtilis*, *S. aureus*	*E. coli*, *P. aeruginosa*, *Salmonella typhimurium*, *Klebsiella pneumoniae*, *Vibrio cholerae*	[65]
*Desmococcus olivaceus*	Ethanolic extracts	*S. aureus*	*E. coli*, *P. aeruginosa*	[51]
*Dunaliella primolecta*	Polyunsatured fatty acids: alpha-linolenic acid	*B. cereus*, *B. subtilis*, *S. aureus*, MRSA	*E. aerogenes*	[53,64]
*Dunaliella salina*	Indolic derivative, polyunsaturated fatty acids, beta-ionone and neophytadiene	*S. aureus*	*E. coli*, *P. aeruginosa*	[52,66,67]
*Dunaliella* sp.	Lysed cells	*S. epidermidis*, *Micrococcus luteus*	*Proteus vulgaris*	[68]
*Haematococcus pluvialis*	Short-chain fatty acids	*S. aureus*	*E. coli*	[69,70]
*Klebsormidium* sp.	Pellet	*B. subtilis*	Ne	[50]
*Pseudokirchneriella subcapitata*	Methanolic extracts	*S. aureus*	*P. aeruginosa*	[52]
*Scenedesmus obliquus*	Long chain fatty acids	*S. aureus*	*E. coli*, *P. aeruginosa*, *Salmonella* sp.	[71]
*Scenedesmus quadricauda*	Various organic solvent extracts	*B. subtilis*, *S. aureus*	*E. coli*, *P. aeruginosa*	[63]
*Scenedesmus* sp.	Ethanolic extracts	*S. aureus*	*E. coli*, *P. aeruginosa*	[51]
**Red microalgae**				
*Porphyridium aerugineum*	Phycobiliproteins	*S. aureus*, *S. pyogenes*	Nt	[72]
*Porphyridium purpureum*	Methanolic extracts	*B. subtilis*	*E. coli*	[50]
*Porphyridium sordidum*	Pellet	*B. subtilis*	*E. coli*, *Pseudomonas fluorescens*	[50]
*Rhodella reticulata*	Exopolysaccharides	*S. aureus*, *B. cereus*, *S. pyogenes*	Ne	[72]
**Diatoms**				
*Asterionella glacialis*	Whole cell	*S. aureus*, *S. epidermidis*, *M. luteus*, *Sarcina* sp.	*E. coli*	[58]
*Attheya longicornis*	Methanolic extracts	*S. aureus*, MRSA	Ne	[73]
*Chaetoceros muelleri*	Unsaturated fatty acid-containing lipidic fractions (triglycerides and docosa-pentaenoic acid (DPA))	*B. subtilis*, *S. aureus*	*E. coli*	[74,75]
*Navicula delognei (Parlibellus delognei)*	transphytol ester, hexadecatetraenoic and octadecatetraenoic acids	*S. aureus*, *S. epidermidis*	*S. typhimurium*, *P. vulgaris*	[76]
*Phaeodactylum tricornutum*	eicosapentaenoic acid (EPA), palmitoleic and hexadecatrienoic acids (HTA)	*B. cereus*, *Bacillus weihenstephanensis*, *S. aureus*, *S. epidermidis*, MRSA	Ne	[77,78]
*Rhizosolenia alata*	Various organic solvent extracts	*B. subtilis*, *S. aureus*	*E. coli*, *P. aeruginosa*, *P. vulgaris*, *S. typhi*, *V. cholerae*	[79]
*Thalassiothrix frauenfeldii*	Non-axenic culture and organic solvent extracts	*B. subtilis*, *S. aureus*	*E. coli*, *P. aeruginosa*, *Salmonella paratyphi*, *S. typhi*, *V. cholerae*	[80]
*Skeletonema costatum*	Aqueous and organic extracts	*B. subtilis*, *S. aureus*	*P. aeruginosa*	[81]
*Skeletonema costatum*	Various organic solvents extracts	*S. aureus*, *Staphylococcus peoria*, *S. fecalis*, *S. pyogenes*	Ne	[54]
**Haptophytes**				
*Isochrysis galbana*	Chlorophyll a derivatives: pheophytin a and chlorophyllide a	*S. aureus*, *Streptococcus faecalis*, *S. pyogenes*, *Micrococcus* sp.	Nt	[54,82]

Ne = No effect of the microalgal compound against the bacteria tested; Nt = Not tested; MRSA = Methicillin resistant *S. aureus*.

**Table 2:** Antibacterial activity from microalgae against diseases in aquaculture.

Microalgae Species	Compound/Fraction Tested	Target Bacteria/Antibacterial Effect	References
**In vitro experiments**			
*Chaetoceros lauderi*	Whole cell	*Vibrio anguillarum*, *Aeromonas salmonicida*	[58]
*Dunaliella tertiolecta*	Aqueous extract	*Vibrio campbellii*	[115]
*Euglena viridis*	Organic solvent extracts	*Aeromonas hydrophila*, *Edwardsiella tarda*, *Pseudomonas aeruginosa*, *Pseudomonas fluorescens*, *Pseudomonas putida Vibrio alginolyticus*, *V. anguillarum*, *Vibrio fluvialis*, *Vibrio harveyi*, *Vibrio parahaemolyticus*	[116]
*Haslea karadagensis*	Purified pigment (intra- and extracellular forms)	*Polaribacter irgensii*, *Pseudoalteromonas elyakowii*, *Vibrio aestuarianus*	[117]
*Haslea ostrearia*	Purified marennine (intra- and extracellular forms)	*P. irgensii*, *P. elyakowii*, *V. aestuarianus*	[118]
	Purified marennine (intracellular form)	*V. anguillarum*	[119]
	Purified marennine (extracellular form)	*Vibrio splendidus*-related	[30]
*Phaeodactylum tricornutum*	Aqueous and organic extracts	*Alcaligenes cupidus*, *Alteromonas communis*, *Alteromonas haloplanktis*, *Vibrio fischeri*, *V. parahaemolyticus*	[81]
	Polyunsaturated free fatty acid	*Vibrio anguillarum*, *M. luteus*, *Photobacterium* sp.	[78]
*Skeletonema costatum*	Aqueous and Organic extracts	*A. cupidus*, *A. communis*, *Pseudomonas marina*, *V. fischeri*, *V. parahaemolyticus*	[81]
	Organic and purified extracts	*V. anguillarum*, *Vibrio mytili T*, *Vibrio* spp. *S322*, *Vibrio* spp. *VRP*	[120]
	Aqueous extracts	*Vibrio campbellii*	[115]
*Stichochrysis immobilis*	Microalgal homogenates	*Xanthomonas* sp. *1*, *Flavobacterium* sp. *1*, *Pseudomonas* sp. *Strain 101*	[121]
*Tetraselmis suecica*	Microalgal supernatant and microalgal homogenates of a commercial spray-dried preparation	*A. hydrophila*, *A. salmonicida*, *Serratia liquefaciens*, *V. alginolyticus*, *V. anguillarum*, *V. parahaemolyticus*, *Vibrio salmonicida*, *Vibrio vulnificus*, *Yersinia ruckeri*	[122,123]
**Co-culture experiments**			
*Chaetoceros calcitrans*	Axenic culture	*V. harveyi*	[124]
*Chlorella minutissima*	Axenic culture	*V. alginolyticus*, *V. anguillarum*, *Vibrio lentus*, *V. parahaemolyticus*, *Vibrio scophthalmi*, *V. splendidus*	[125]
*Chlorella* sp.	Axenic culture	*V. harveyi*	[40]
*Isochrysis galbana*	Non-axenic culture	*V. alginolyticus*, *V. campbellii*, *V. harveyi*	[126]
*Isochrysis* sp.	Axenic culture	*V. alginolyticus*, *V. lentus*, *V. splendidus*, *V. scophthalmi*, *V. parahaemolyticus*, *V. anguillarum*	[125]
*Nannochloropsis* sp.	Axenic culture	*V. alginolyticus*, *V. anguillarum*, *V. lentus*, *V. parahaemolyticus*, *V. scophthalmi*, *V. splendidus*	[125]
*Nitzchia* sp.	Axenic culture	*V. harveyi*	[124]
*S. costatum*	Exometabolites in the culture fluid	*Listeria monocytogenes*	[127]
	Axenic culture	*Pseudomonas* sp., *Vibrio* sp.	[128]
*Tetraselmis chui*	Axenic culture	*V. alginolyticus*, *V. anguillarum*, *V. lentus*, *V. parahaemolyticus*, *V. scophthalmi*, *V. splendidus*	[125]
**In vivo experiments**			
*C. minutissima*	30 min incubation of enriched *Artemia metanauplii*	Decrease of the bacterial load in *Artemia* and diminution of presumptive *Vibrio*	[129]
*D. tertiolecta*	Daily diet of *Artemia franciscana*	Protection against *V. campbellii* and *V. proteolyticus*	[39]
*H. ostrearia*	Incubation of *Mytilus edulis* larvae with supernatant containing extracellular pigments	Higher survival and physiological conditions of larvae challenged with *V. splendidus*-related	[130]
*Tetraselmis* sp.	4 h incubation of *Artemia franciscana*	Diminution of associated bacteria, better bacterial diversity and the flora less dominated by *V. alginolyticus*	[131]
*T. suecica*	Food supplement for the Atlantic salmon *Solmo salar*	Reduction of *A. hydrophila*, *A. salmonicida*, *Serratia liquefaciens*, *V. anguillarum*, *V. salmonicida*, *Y. ruckeri* infections, reduction of bacterial populations in water tanks and increase of the microbial communities in the digestive tract	[123]
	Food supplement for the broodstock and partial live larvae feed for the white prawn *Fenneropenaeus indicus*	Reduction of *Vibrio* numbers in the water tank, better egg hatching rate and survival rate of the larvae	[132]

**Table 3:** Antifungal activity from microalgae.

Microalgae Species	Antifungal Compounds/Fraction	Target Microorganims	References	
**Green microalgae**				
*Chlorella vulgaris*	Microalgal supernatant	Yeast: *Candida kefyr* Mold: *Aspergillus fumigatus*, *Aspergillus niger*	[62]	
*Chlorococcum humicola*	Organic solvent extracts and pigments: beta carotene, Chlorophyll a and Chlorophyll b	Yeast: *C. albicans* Mold: *A. flavus*, *A. niger*	[65]	
*Heterochlorella luteoviridis*	Microalgal supernatant	Yeast: *C. albicans*	[50]	
*Haematococcus pluvialis*	Short-chain fatty acids	Yeast: *C. albicans*	[70]	
*Scenedesmus quadricauda*	Organic solvent extracts	Yeast: *C. albicans*, *S. cerevisiae* Mold: *A. flavus*, *A. niger*, *P. herquei* Other: *A. brassicae*, *F. moniliforme*, *Helminthosporium* sp.	[63]	
**Red microalgae**				
*Porphyridium aerugineum*	Phycobiliproteins	Yeast: *C. albicans*	[72]	
*Porphyridium purpureum*	Microalgal supernatant	Yeast: *C. albicans*	[50]	
*Rhodella reticulata*	Exopolysaccharides	Yeast: *C. albicans*	[72]	
**Diatoms**				
*Chaetoceros lauderi*	Polysaccharides	Mold: *A. fumigatus*, *Blastomyces dermatitidis*, *Emmonsia parva*, *Madurella mycetomi*, *Sporothrix schenckii* Dermatophyte: *Epidermophyton floccosum*, *Microsporum audouini*, *Microsporum canis*, *Microsporum ferrugineum*, *Microsporum gypseum*, *Microsporum nanum*, *Microsporum persicolor*, *Trichophyton* spp.	[58,162,163]	
*Chaetoceros muelleri*	Lipidic fractions: triglycerides, docosapentaenoic acid (DPA)	Yeast: *C. albicans*	[75]	
*Haslea karadagensis*	Purified pigment (intra- and extracellular forms)	*Corollospora maritima*, *Lulworthia* sp., *Dendryphiella salina*	[117]	
*Thalassiothrix frauenfeldii*	culture filtrates and organic solvent extracts	Yeast: *C. albicans*, *Candida glabrata*, *Candida krusei*, *Candida tropicalis*, *Cryptococcus neoformans* Mold: *A. niger*	[80]	
**Dinoflagellates**				
*Amphidinium* sp.	Polyols: karatungiols A(1)	Mold: *A. niger*	[164]	
*Gambierdiscus toxicus*	Gambieric acids A and B forms	Mold: *A. fumigatus*, *A. niger*, *Aspergillus oryzae*, *Penicillium citrinum*, *Penicillium chrysogenum*, *Paecilomyces variotii* Dermatophyte: *E. floccosum*, *T. mentagrophytes*	[165]	
*Goniodoma pseudogonyaulax*	Goniodomin A (polyether macrolide)	Yeast: *C. albicans*, *C. neoformans*, *S. cerevisiae* Mold: *Penicillium* sp. Dermatophyte: *T. mentagrophytes*	[166,167]	
*Prorocentrum lima*	Polyethers	Yeast: *Candida rugosa* Mold: *A. niger*, *Penicillium funiculosum*	[159]	

**Table 4:** Antiviral activity from microalgae.

Microalgae Species	Antiviral Compound and Cytotoxicity (μg/mL)	Target Virus	Mechanism of Action and Efficiency (μg/mL)	References
**Green microalgae**				
*Chlorella vulgaris*	Polysaccharide-rich fraction CC_50_ > 1600 (Vero cells)	HSV-1	Inhibits attachment, replication	[201]
			IC_50_ = 61	
*Dunaliella primolecta*	Pheophorbide-like compound Not cytotoxic (Vero cells)	HSV-1	Inhibits adsorption, invasion	[202]
			MIC = 5 (totally inhibit the CPE)	
*Dunaliella salina*	Short chain fatty acids, β-ionone, neophytadiene, phytol, palmitic and α-linolenic acids CC_50_ = 1711 (Vero cells)	HSV-1	Inhibits infectivity	[203]
			IC_50_ = 85	
*Haematococcus pluvialis*	Polysaccharide-rich fraction CC_50_ = 1867 (Vero cells)	HSV-1	Inhibits attachment, penetration, replication	[203]
			IC_50_ = 99	
**Red microalgae**				
*Porphyridium cruentum*	Sulphated exopolysaccharide Not cytotoxic at 100 (HeL cells)		Inhibits penetration, replication	[204]
		HSV-1	EC_50_ (HSV-1) = 34	
		HSV-2	EC_50_ (HSV-2) = 12	
		Vaccina	EC_50_ (Vaccina) = 12	
*Porphyridium purpureum*	Exopolysaccharide Not cytotoxic at 500 (HEp-2 cells)	Vaccina	Interaction with free viral particles	[205]
			IC_50_ = 0.65	
*Porphyridium* sp.	Sulphated polysaccharide Not cytotoxic at 250 (Vero cells) and 2000 (in vivo in rats)	HSV-1	In vitro: inhibits adsorption, replication	[200,206]
			CPE_50_ = 1	
			In vivo: prevents the development of symptoms at 100	
			Inhibits adsorption, replication	
		HSV-2	CPE_50_ (HSV-2) = 5	
		VZV	CPE_50_ (VZV) = 0.7	
*Porphyridium* sp.	Purified polysaccharide Not cytotoxic at 1000 (NIH/3T3 cells)	MuSV/MuLV	Inhibits the production of retroviruses in the cells	[207]
			RT_50_ reduction = 5	
		MuSV-124	Inhibits cell transformation	
			ffu_50_ protection = 10	
**Diatoms**				
*Haslea karadagensis*	Purified pigment: intra- and extracellular forms	HSV-1	Inhibits infection, cell destruction	[117]
	CC_50_ (Int) = 87		EC_50_ (Int) = 62	
	CC_50_(Ext) > 200 (Vero cells)		EC_50_ (Ext) = 23	
*Haslea ostrearia*	Purified pigment: intra- and extracellular forms	HSV-1	Inhibits infection, cell destruction	[117]
	CC_50_ (Int) > 200 (Vero cells)		EC_50_ (Int) = 24	
	CC_50_ (Ext) = 107 (Vero cells)		EC_50_ (Ext) = 27	
	Water soluble extract CC_50_ > 200 (Vero and MT-4 cells)	HSV-1	Inhibits replication	[208]
			EC_50_ = 14	
*Navicula directa*	Sulphated polysaccharide: Naviculan		Inhibits adhesion, penetration	[209]
	CC_50_ (HSV-1) = 3800 (Vero cells)	HSV-1	IC_50_ (HSV-1) = 14	
	CC_50_ (HSV-2) = 3800 (Vero cells)	HSV-2	IC_50_ (HSV-2) = 7.4	
	CC_50_ (IFV-A)= 5400 (MDCK cells)	IFV-A	IC_50_ (IFV-A) = 170	
	CC_50_ (HIV-1) = 4000 (HeLA cells)	HIV-1	IC_50_ (HIV-1) = 53	
**Dinoflagellates**				
*Cochlodinium polykrikoides*	Extracellular sulphated polysaccharides: A1 and A2		Inhibits replication and the CPE	[210]
	CC_50_ (HIV-1) > 100 (MT-4 cells)	HIV-1	IC_50_ (HIV-1) = 1.7	
	CC_50_ (IFV-A) > 100 (MDCK cells)	IFV-A	IC_50_ (IFV-A) = 0.45–1	
	CC_50_ (IFV-B) > 100 (MDCK cells)	IFV-B	IC_50_ (IFV-B) = 7.1–8.3	
	CC_50_ (RSV-A) > 100 (Hep-2 cells)	RSV-A	IC_50_ (RSV-A) = 2–3	
	CC_50_ (RSV-B) > 100 (Hep-2 cells)	RSV-B	IC_50_ (RSV-B) = 0.8	
	A1	HSV-1	IC_50_ = 4.5	
	CC_50_ > 100 (HMV-2 cells)			
	A2	PFluV-2	IC_50_ = 0.8	
	CC_50_ > 100 (HMV-2 cells)			
*Gyrodinium impudicum*	Purified sulphated exopolysaccharide: p-KG03	EMCV	Inhibits the development of the CPE, suppress tumor cell growth EC_50_ = 27	[211]
	CC_50_ = 3.4 (MT-4 cells)			
	CC_50_ = 59.9 (Vero cells)			
	CC_50_ > 1000 (HeLa cells)			
	Not in MDCK cells CC_50_ > 100		Inhibits adsorption	[212]
		IFV-A	EC_50_ (IFV-A) = 0.19–0.48	
		IFV-B	EC_50_ (IFV-B) = 0.26	

EMCV: encephalomyocarditis virus; HIV-1: human immunodeficiency virus type 1; HSV-1: Herpes simplex virus type 1; HSV-2: herpes simplex virus type 2; IFV-A: influenza A virus; IFV-B: influenza B virus; MuLV: murine leukemia virus; MuSV-124: murine sarcoma virus; RSV: respiratory syncytial virus; VZV: varicella zoster virus. CC_50_: concentration that kills 50% of the infected cells; CPE_50_: concentration that offers 50% protection against the cytopathic effect; EC_50_: concentration requires to inhibit 50% of the virus-induced cytopathic effects (CPE); ffu_50_: concentration that offers 50% protection against the formation of foci of malignant cells; IC_50_: concentration that inhibits 50% of the virus infection; MIC: minimum inhibitory concentration; RT_50_: concentration that offers 50% reduction of reverse transcriptase activity.
